# Reliability Exercise for the Polymyalgia Rheumatica Classification Criteria Study: The Oranjewoud Ultrasound Substudy

**DOI:** 10.1155/2009/738931

**Published:** 2009-06-14

**Authors:** Alexander K. Scheel, Eric L. Matteson, Bhaskar Dasgupta, George A. W. Bruyn, Sarah Ohrndorf, Carola Werner, Wolfgang A. Schmidt

**Affiliations:** ^1^Department of Nephrology and Rheumatology, Georg-August-University Göttingen, 37073 Göttingen, Germany; ^2^Department of Rheumatology, Johann Wolfgang Goethe-University, Theodor Stern Kay 7, 60590 Frankfurt am Main, Germany; ^3^Division of Rheumatology, Mayo Clinic College of Medicine, Rochester, MN 55905, USA; ^4^Department of Rheumatology, Southend University Hospital, Westcliff on Sea, Essex SSO-ORY, UK; ^5^Department of Rheumatology, Medisch Centrum Leeuwarden, 8934 Leeuwarden, The Netherlands; ^6^Institute of Medical Statistics, Georg-August-University Göttingen, 37073 Göttingen, Germany; ^7^Medical Centre for Rheumatology Berlin-Buch, 13125 Berlin, Germany

## Abstract

*Objective*. A study supported by the EULAR and the ACR being conducted to establish classification criteria for polymyalgia rheumatica (PMR) will include ultrasound examination of the shoulders and hips. Ultrasound (US) depicts glenohumeral joint effusion, biceps tenosynovitis, subdeltoid bursitis, hip joint synovitis, and trochanteric bursitis in PMR. These findings may aid in distinguishing PMR from other diseases. The purpose of this study was to assess standards and US interreader agreement of participants in the PMR classification criteria study. *Methods*. Sixteen physicians in four groups examined shoulders and hips of 4 patients and 4 healthy adults with ultrasound. Overall agreement and interobserver agreement were calculated. *Results*. The overall agreement (OA) between groups was 87%. The OA for healthy shoulders was 88.8%, for healthy hips 100%, for shoulders with pathology 85.2%, and 74.3% for hips with pathology, respectively. *Conclusion*. There was a high degree of agreement found for the examination of healthy shoulders and pathologic hips. Agreement was moderate for pathologic shoulders and perfect for healthy hips. US of shoulder and hips performed by different examiners is a reliable and feasible tool for assessment of PMR related disease pathology and can be incorporated into a classification criteria study.

## 1. Introduction

Polymyalgia rheumatica (PMR) is the most common inflammatory rheumatic disease in the elderly. There is considerable uncertainty related to diagnosis and outcomes in patients presenting with the polymyalgic syndrome. Therefore the European League against Rheumatism (EULAR) and the American College of Rheumatology (ACR) are supporting a study that is being conducted to establish classification criteria for PMR [1]. Ultrasound (US) of shoulders and hips was selected among several other candidate criteria that had been recruited by a Delphi survey from a work group of 27 international physicians with interest in PMR (rheumatologists, nonrheumatologists, statisticians, and methodologists) who met to pursue a consensus-based process for the development of classification criteria in PMR in Cambridge, UK, in 2005. Participants agreed that currently, there was no role for routine use of magnetic resonance imaging or positron emission tomography in the evaluation of suspected PMR. However musculoskeletal US was thought to have utility as a diagnostic criterion for PMR due to widespread availability, feasibility, and results of preliminary studies in this condition [2–7].

US depicts characteristic pathologic findings of shoulder joints and the hip region that may aid in distinguishing PMR from other diseases that may mimic it. Typical findings on US include glenohumoral joint synovitis, subdeltoid bursitis, and biceps tendon tenosynovitis of the shoulders [2–6]. These findings occur in most PMR patients, but they are often mild in contrast to elderly onset rheumatoid arthritis (EORA). In the hips, US often reveals hip joint synovitis and trochanteric bursitis in patients with PMR [2, 5, 7].

Before considering US as a potential tool for the classification, there was agreement regarding the need to standardise the examination and assess the inter-observer agreement for distinguishing lesions typical of PMR from other lesions like extensive effusion or bursitis, rotator cuff tears, rotator cuff calcifications, and osteoarthritis.

To pursue this aim, members of centres participating in the PMR classification criteria study met to standardise the US examination in PMR patients for each centre and work towards achieving a high degree of inter-reader agreement in the US examination for the PMR classification criteria study. The study included both, patients with PMR and other related diseases with shoulder pain such as rheumatoid arthritis (RA). While we present a validation study, our study does not address the question of defining US characteristics of PMR.

## 2. Methods

### 2.1. Study Protocol

Fifteen rheumatologists and one radiologist of varying US experience from the participating centres (listed in the Acknowledgement) met in Oranjewoud (The Netherlands) to evaluate the possibility of including US in the upcoming PMR classification criteria study. The meeting took place just following the 12th EULAR basic ultrasound course and before the annual EULAR congress in June 2006. The 16 physicians were assigned to 4 groups. Each group consisted of four assessors: one supervisor, one performing the US scans, one measuring distances between structures, and one documenting the results. The ultrasound examiners were—with the exception of the supervisor—alumni of the basic EULAR sonography course. None were career specialists in the field of musculoskeletal ultrasonography. Prior to the patient examination, each supervisor gave a review of the US examination including specifics of the standard scans to be performed for operator training. Standard definitions and pathologic findings were demonstrated. The group as a whole decided if they regarded the US findings as normal or abnormal. To ensure standardised documentation, each participant was given a report sheet that listed possible pathologic findings recorded as “yes” or “no,” indicating the presence or absence of each particular finding. All US assessments were performed independently by each group without contact with other groups and without knowledge of the patient's disease or joint status. Each group was given a maximum of 15 minutes (shoulder joint) and 10 minutes (hip joint), respectively, for US examination per joint region, rotating on a preset plan from one US station to the next. Each healthy control and each patient was assigned to every US station in rotation.

The focus of the US examinations was the shoulder and hip joints. Two joints (one shoulder and one hip) were examined in 4 healthy controls and in 4 patients with RA or PMR. Four rounds (normal shoulder, normal hip, pathologic shoulder, and pathologic hip) for 4 groups were necessary to ensure that an examination was performed by each group, control/patient and joint region. Patients and healthy individuals were recruited from the Medisch Centrum Leeuwarden by GAWB. All patients gave their consent to participation in the study. Subject characteristics were as in Tables 1 and 2.

### 2.2. Ultrasonography

Examinations were performed with several US machines which are in use in practice and simulate the conditions of the PMR classification study. A first phase showed a good reliability between different US machines [8]. Stations 1 and 2 were equipped with a Mylab 70 (Esaote, Genoa, Italy). Shoulder and hip joints were examined with a linear array probe (LA 18–6 MHz), respectively. Station 3 was equipped with a Logic E (General Electrics, Milwaukee, USA), and Station 4 with a Voluson I (General Electrics, Milwaukee, USA). Shoulder and hip joints were examined with a linear array probe (12L-RS, 13–5 MHz), respectively. Two equipment specialists each from Esaote and General Electrics were present to help in case of problems with regard to machine adjustments during the examinations.

Scanner settings were uniform for all measurements: frequency setting, B-mode gain, and 100%; one focus point position in the region of measurement. An introduction to the US device was given to the observers prior to US examinations.

Standard scans according to the EULAR guidelines for musculoskeletal US were applied [9]. For the shoulder joint, examination of the biceps tendon, rotator cuff (subscapularis, supraspinatus, and infraspinatus tendons), glenohumeral joint, acromioclavicular joint, humeral bone surface subacromial-subdeltoid bursa, and axillary artery was required. Effusion, synovitis, tenosynovitis, bone erosions, osteophytes, and bursitidies were evaluated. Evaluation of the hip joints included assessment for effusion, synovitis, osteoarthritis, and trochanteric bursitis.

Synovitis, effusion, tenosynovitis, and erosions were defined according to the OMERACT definitions for musculoskeletal ultrasound [10].

### 2.3. Statistical Analysis

Overall agreement and inter-observer agreement values were calculated. All analyses were calculated with Statistical Product and Service Solutions (SPSS) 15.0 (Chicago, Ill, USA).

## 3. Results

The overall agreement between the 4 groups of sonographers with regard to all normal and pathologic findings for shoulders and hips was 87%, reflecting substantial agreement.

For the healthy controls, overall agreement for the shoulder was 88.8% (substantial agreement). For the hip joint, overall agreement was 100% (perfect agreement). For the patient group, overall agreement for the shoulder joint was 85.2% (moderate agreement). For the pathologic hip joint, overall agreement was 74.3% (substantial agreement).

**Figure 1:**
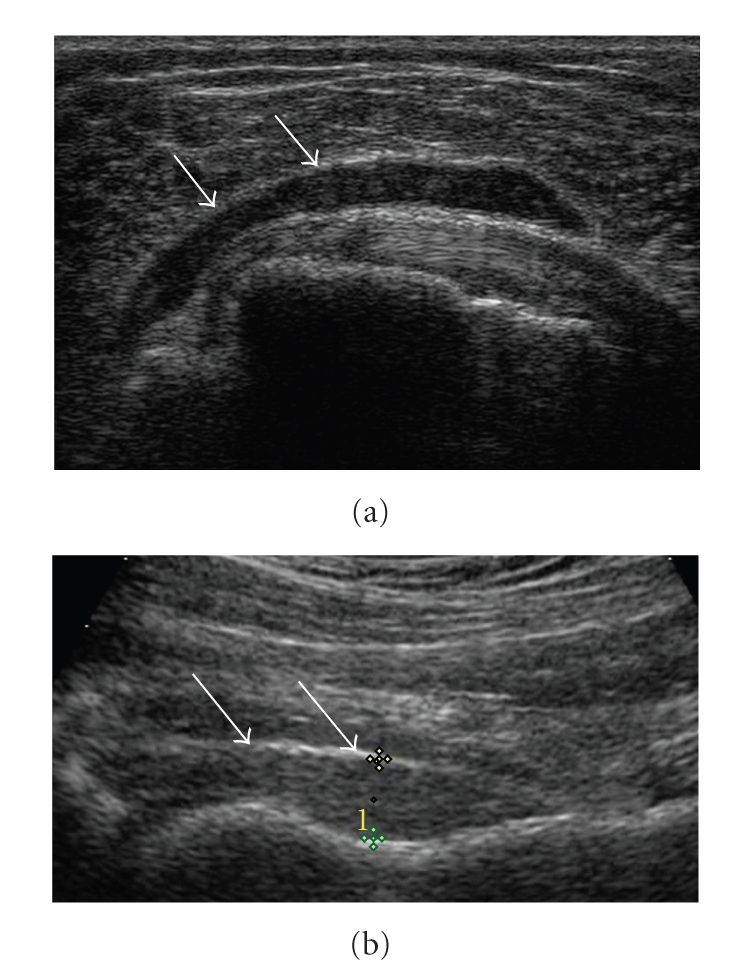
(a) Subdeltoid bursitis (arrows) in a transverse view of the shoulder. (b) Longitudinal view of hip joint synovitis. The joint capsule (short arraw) is not parallel to the bone surface of the femur (long arrow). The distance between bone and joint capsule is pathologic (>8 mm).

The overall agreements with regard to the different pathologies for the shoulder and hip joints are displayed in Tables 3 and 4. Figures 1(a) and 1(b) show typical joint pathologies in PMR patients, for example, subdeltoid bursitis and hip joint synovitis.

## 4. Discussion

US plays an important role in the detection of many inflammatory processes. It is important to rigorously evaluate the utility of US in PMR since there are no recognised laboratory or imaging diagnostic tests for the condition. Recent studies have shown the importance of US in depicting characteristic pathologies that aid in distinguishing PMR from other mimicking diseases. The most frequent US soft tissue alterations in patients with PMR have been described for the shoulders (subdeltoid bursitis, tenosynovitis of the biceps tendon, and glenohumeral synovitis) [2–6] and for the hips (synovitis and trochanteric bursitis) [2, 5, 7].

However, to date US has not yet been included in any diagnostic or classification criteria for PMR. Therefore, a study group has met to evaluate the possibility of including US in the upcoming PMR classification criteria study. US examinations were performed according to the EULAR criteria [9]. Our standardized protocol and study results demonstrated that US examinations for both, shoulder and hip joints can be a useful tool for assessment of pathology in a future study of PMR; since it can be performed by competent examiners across centres with reliable result.

Some former studies have investigated the prevalence for the detection of inflammatory changes of the shoulder [2–6] as well as the hip [2, 5, 7] joints in PMR by US. Cantini et al. performed a case control study of shoulder US in PMR patients including 57 consecutive patients with PMR and 114 controls with bilateral shoulder pain and stiffness due to RA, psoriatic arthritis, spondyloarthritis, osteoarthritis, fibromyalgia, or connective tissue disease. Twenty-four PMR patients were also examined with MRI. Bursitis was detected in all patients, glenohumeral synovitis in 88%, and biceps tendon tenosynovitis in 88% [7]. Other authors have reported that inflammatory changes are discrete in PMR but much more severe in EORA [3]. Frediani et al. [5] performed a case control study that describes the US findings in 50 consecutive patients with PMR, spondyloarthritis, and RA, respectively. They detected biceps tenosynovitis in 44% of spondyloarthritis patients and in 38% of patients with RA, glenohumoral joint synovitis in 16% of patients with spondyloarthritis, and in 54% of patients with RA, and subdeltoid bursitis in 34% of patients with spondyloarthritis and in 44% of patients with RA.

Since a high prevalence of synovitis is seen in shoulder and hip joints of PMR patients, the current study focused on whether overall agreement could be achieved between readers for typical joint pathologies seen in PMR patients (including subdeltoid bursitis, biceps tenosynovitis, and glenohumoral synovitis for the shoulder joint, resp.). We found substantial overall agreement for detecting these pathologies in normal as well as pathologic shoulder and hip joint pathologies. These findings underline the fact that US is not as observer dependent as formerly thought [11]. Furthermore, our results demonstrated the feasibility of performing a multicentre study using US for evaluation of patients with shoulder and hip lesions typical of PMR.

US is an important imaging tool for the visualisation of inflammation in shoulders and hips joints of PMR. In this exercise substantial agreement was found for the examination of healthy shoulders and pathologic hips. Agreement was moderate for pathologic shoulders and perfect for healthy hips. The results of this study confirm that US can be evaluated as a diagnostic technique in studies of PMR including the PMR classification criteria study.

## Figures and Tables

**Table 1:** 

Healthy individuals' characteristics		Part examined	
Male, 36 years	No shoulder or hip pain	Right shoulder	Right hip
Male, 48 years	No shoulder or hip pain	Right shoulder	Right hip
Female, 46 years	No shoulder or hip pain	Left shoulder	Left hip
Female, 29 years	No shoulder or hip pain	Left shoulder	Left hip

**Table 2:** 

Patients' characteristics	
Female, 64 years	Symptomatic right shoulder, left hip, RA (since 1999)
Female, 48 years	Symptomatic right shoulder, right hip, RA (since 1990)
Female, 79 years	Symptomatic right shoulder, left hip, temporal arteritis with PMR since 2003
Female, 75 years	Symptomatic left shoulder, right hip, PMR, aortitis since 2005

**Table 3:** Overall agreements for normal and pathologic shoulders.

Structure	Pathology	Overall agreement normal shoulder	Overall agreement pathologic shoulder
Biceps tendon	Tenosynovitis	100%	75%
	Rupture	87.5%	100%
Subdeltoid bursa	Bursitis	87.5%	87.5%
Glenohumeral joint	Synovitis/effusion at posterior joint space	75%	75%
Glenohumeral joint	Synovitis/effusion at axillary recess	75%	100%
Subscapularis tendon	Complete rupture	100%	100%
	Partial rupture	87.5%	100%
	Calcification	87.5%	87.5%
Supraspinatus tendon	Complete rupture	100%	87.5%
	Partial rupture	83.3%	70.8%
	Calcification	87.5%	54.2%
Infraspinatus tendon	Complete rupture	100%	100%
	Partial rupture	87.5%	100%
	Calcification	100%	100%
Rotator cuff	Impingement	87.5%	100%
Acromioclavicular joint	Osteoarthritis (osteophytes)	62.5%	41.6%
	Synovitis/effusion	62.5%	50%
Humeral head	Erosion	83.3%	70.8%
Axillary artery	Vasculitis	100%	100%
	Arteriosclerotic plaques	100%	75%
	Stenosis >50%	100%	100%
	Occlusion	100%	100%

**Table 4:** Overall agreements for normal and pathologic hips.

Structure	Pathology	Overall agreement normal Hip	Pathology	Overall agreement pathologic Hip
Hip joint	Effusion/synovitis	100%	Effusion/synovitis	88%
Hip joint	Osteoarthritis (osteophytes)	100%	Osteoarthritis (osteophytes)	75%
Trochanteric bursa	Bursitis longitudinal	100%	Bursitis longitudinal	70.8%
	Bursitis transverse	100%	Bursitis transverse	66.7%
	Bursitis sagittal	100%	Bursitis sagittal	70.8%
